# 

**Published:** 1914-01

**Authors:** 


### CONTENTS.

ORIGINAL COMMUNICATIONS—

A Method of Easily Effecting the Solution of Salvarsan and a Comparison of the Value of Salvarsan and Neo-Salvarsan, by John Banks, A. B., M. D., LaGrange, Ga. .......... 431

Some Rambling Thoughts on Medical Europe, by Dunbar Roy, A. B., M. D., Atlanta, Ga. .......... 436

Report of One Hundred and Forty-Eight Herniotomies With Description of Operation, by L. C. Fischer, M. D., Atlanta, Ga. .......... 442

Heredity In Nervous Diseases and Its Social Bearings, by W. A. Gardner, M. D., Atlanta, Ga. .......... 456

Fulton County Medical Society, Regular Meeting, November 20, 1913, by R. R. Daly, M. D. .......... 461

Vagitus Uterinus, Report of Two Cases, by F. J. Giuffrida, Chief of Out-Patient Obstetrical Department of the Atlanta Medical School, Atlanta, Ga. .......... 471

Eugenics .......... 474

Program of Clinics Arranged for the Georgia Surgeons' Club by the Surgeons of New Orleans, La., February 27-28, 1914 .......... 477

